# ChatGPT to Enhance Learning in Dental Education at a Historically Black Medical College

**DOI:** 10.26502/droh.0069

**Published:** 2024-01-25

**Authors:** Khandoker Rahad, Kianna Martin, Ihunna Amugo, Shania Ferguson, Angela Curtis, Anniya Davis, Pandu Gangula, Qingguo Wang

**Affiliations:** 1 Department of Computer Science & Data Science, School of Applied Computational Sciences, Meharry Medical College, Nashville, TN, USA; 2 Department of ODS & Research, School of Dentistry, Meharry Medical College, Nashville, TN, USA

**Keywords:** Large language model, ChatGPT, Dentistry, Dental Education, Student Learning

## Abstract

The recent rise of powerful large language model (LLM)-based AI tools, exemplified by ChatGPT and Bard, poses a great challenge to contemporary dental education. It simultaneously offers a unique resource that potentially complements today’s teaching and learning, where existing widely available learning resources have often fallen short. Although the LLM tools will shape both the clinical and educational aspects of dentistry profoundly, the didactic curricula, which primarily rely on lecture-based courses where instructors impart knowledge through presentations and discussions, need to be upgraded urgently. In this paper, we used dental course materials, syllabi, and textbooks adopted currently in the School of Dentistry (SOD) at Meharry Medical College to assess the potential utility and effectiveness of ChatGPT in dental education. We collected the responses of the chatbot to questions as well as students’ interactions with it for assessment. Our results showed that ChatGPT can assist in dental essay writing and generate relevant content for dental students, in addition to other benefits. The limitations of ChatGPT were also discussed in the paper.

## Introduction

Large language model (LLM)-based AI chatbots, exemplified by ChatGPT (Chat Generative Pre-Trained Transformer) [1] and Google Bard [2], are remarkably capable of generating human-like responses across a wide spectrum of topics and text formats: formal, informal, content, and creative writing, etc. They pose both challenges and opportunities for the world of education [3–6]. In the context of computer language courses, researchers have harnessed their potential to provide valuable support, including debugging assistance, bug prediction and explanation, contributing to the resolution of programming challenges [5,6]. Moreover, they offer suggestions and code corrections, drawing upon their comprehension of the intricate relationships between code and bugs [5,6]. Comparing with other educational resources, ChatGPT and Bard demonstrate clear advantages in terms of cost, accuracy, customization, user-friendliness, seamless integration with existing tools, and scalability.

Firat et al. explored the integration of ChatGPT in Learning Management Systems (LMS), platforms for delivering and managing online learning content and experiences [7]. ChatGPT enables students to quickly generate answers or essays with minimal efforts, presenting a challenge for instructors. Additionally, it occasionally generates fabricated contents or provides inaccurate answers to certain topics. To manage its risk [8], therefore, it is urgent for contemporary LMS to provide educators with tools to distinguish AI-generated text from human-generated content [8]. Another prominent concern is that the chatbot’s knowledge is limited to its training data, rendering it unaware of the latest events [9]. Instructors, however, may harness this limitation by requiring students to incorporate current events into their writing assignments.

LLMs do not present the same challenges in dentistry as they do in other academic disciplines, because dental schools primarily use oral exams, practical assessments, and supervised patient treatments to evaluate students [10,11]. In fields that rely heavily on written assignments, including essay, thesis, or scientific paper writing, however, curriculum update at dental institutions is inevitable [10,11]. LLMs and other AI applications need to be carefully assessed, regulated, managed, and monitored to ensure their safe, ethical, and beneficial uses in dentistry [10,12]. Moreover, although the practical/clinical aspect of dental education is less affected by LLMs than the pedagogical aspect, the significant potential of LLMs for advancing clinical applications should not be underestimated. For instance, LLM-empowered virtual assistants can personalize not only learning content according to individual needs, but also the communication with patients [11,12]. This will help enhance educational/medical efficiency and reduce the corresponding costs.

To fully realize the potential benefits of LLMs for dental education, the limitations and accuracy of LLMs needs to be assessed, so that effective quality control can be designed and implemented to safeguard against inaccurate and biased responses to dental-related queries [10]. Despite many analyses conducted to assess LLMs’ responses [11–13,20,21], the research in this area is still in its infancy, and the implications of LLMs for dental education remain poorly understood. In this paper, we explore the utility and effectiveness of LLMs to enhance dental students’ learning experiences, with a focus on the use of ChatGPT [1], one of the leading LLMs.

This study was conducted in the School of Dentistry (SOD) at Meharry Medical College (MMC). MMC, as a historically black college/university (HBCU) and the first medical school for African Americans (AA) in the South, has trained over 40% of America’s AA dentists and produced 8% of the nation’s AA physicians. Notably, the majority of dental students and residents in MMC’s SOD are AA (constituting 80% of the student body) and three out of every four of them provide medical or dental services in urban or rural underserved communities. By leveraging ChatGPT to assist dental students in learning, this project can potentially enhance students’ academic performance, which, in turn, will empower MMC to better serve patients in underserved communities. Furthermore, this project was supported in part by a NIMHD supplement grant under award number U54MD007586 [14–16].

## Materials and Methods

This study provides an exploratory assessment of ChatGPT’s utilities for enhancing pedagogical aspect of dental education. To evaluate how well ChatGPT can aid dental students in writing, we collected student essays and then embedded in them incorrect dental terminologies, e.g., wrong word order, improper capitalization and abbreviation, and the use of numerals in place of Roman numerals, etc. The essays were presented to ChatGPT to check if it can recognize and rectify these dental-specific terms. The dental terminologies used in the assessment were selected based on their prevalence in dental curricula. The essays used did not include any patient identifier-patient-related data was not used in any other experiment, either.

Another type of data employed to assess ChatGPT comprises the test objectives of the National Board Examinations (INBDE), which was designed to aid dental boards in determining the qualifications of those seeking licensure to practice dentistry or dental hygiene. ChatGPT-generated outputs based on the INBDE codes for various dental subjects were extensively evaluated by domain experts. ChatGPT was also tasked with responding to a diverse range of queries, including dental students’ assignments such as quizzes, dental literature-related questions, etc.

**Figure 1:**
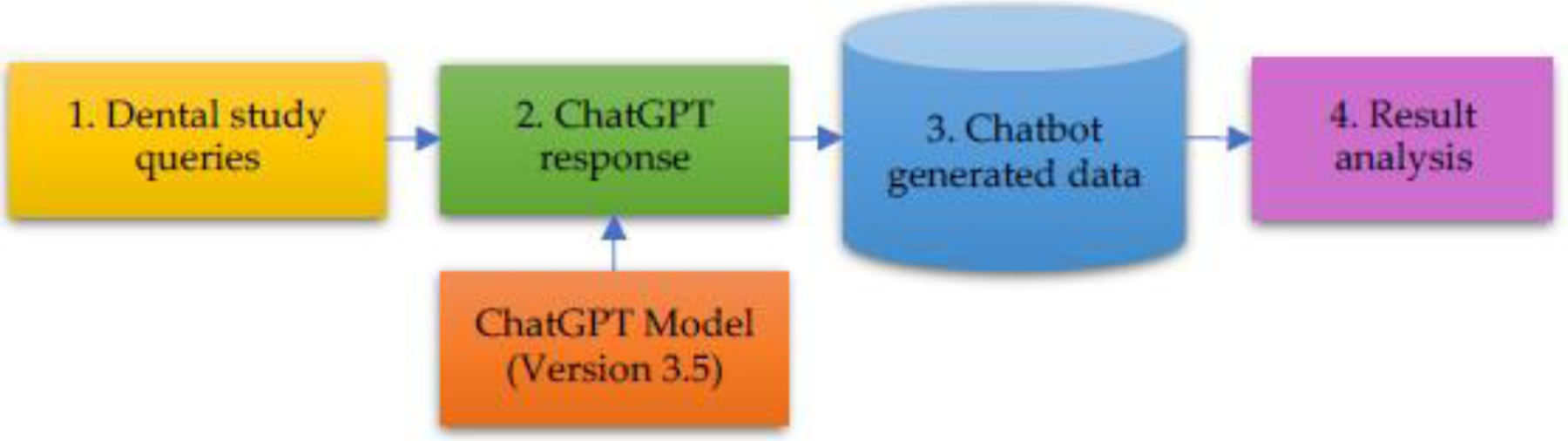
Visualization of data collection and analysis steps.

This study was conducted using the free version of ChatGPT, i.e., version 3.5, which was available at the time when this project started, and which was the one consistently utilized throughout this project. Figure 1 provides an overview of our procedures for collecting and processing the data generated by ChatGPT. These steps are elaborated below:

1. Dental study queries were created based on various categories of assessments in dental education.
2. Dental queries were executed using ChatGPT’s web interface.
3. ChatGPT generated responses were collected for downstream analysis.
4. The collected data was analyzed.

Six domain experts, who also serve as co-authors of this paper, were responsible for collecting data, generating questions for ChatGPT, and verifying its responses. This group included five dental students and a professor from the School of Dentistry. For ChatGPT generated results, the experts employed a Likert scale to assess their accuracy quantitatively. On the data captured, descriptive analyses, including means, median, and frequencies, were performed.

To ensure reproducibility of the work, we validated some selected results. After conducting an initial experiment, we also asked similar set of questions a week later to check the robustness of ChatGPT’s responses. In addition, we followed the guideline as outlined in [17] in all our experiments, the results of which were presented in section below.

## Results

### ChatGPT to enhance writing in dental education

Essays and reflective writings are an important assessment tool in dental curriculum. They are used widely as a means to develop students’ critical thinking skills and communication skills. Due to the fact that ChatGPT’s ability to produce creative and content writing being well documented in other fields [20,21], and to assess the effectiveness of ChatGPT to assist dental students in writing; here we focused on evaluating its capability of recognizing and correcting dental terminologies in student essays.

We collected essays from a group of dental students that describe dental histories of patients. Various types of errors that are commonly found in essays of dental students were added into the essays, such as incorrect alphabet order, improper capitalization and abbreviation, and numerals instead of Roman numerals. Table 1 shows some example errors, from which we can see that many capitalization errors, punctuation errors, spelling errors, subject-verb agreement errors, and other dental-specific errors require dental background to comprehend.

**Figure 2:**
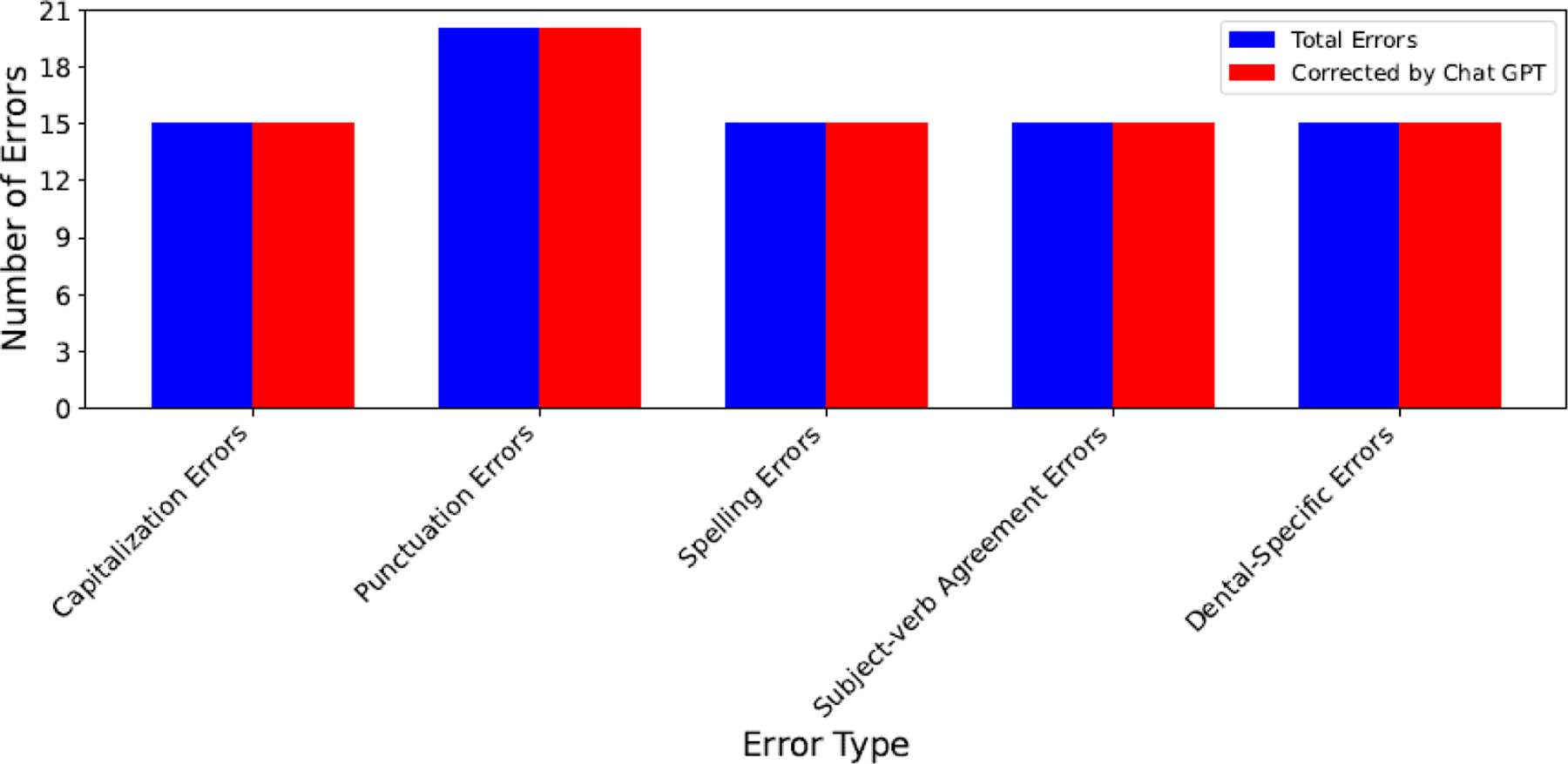
Various errors corrected by ChatGPT across essays on patients’ dental history.

We applied ChatGPT to the essays that contains artificially added errors to check if it can identify and rectify these errors. Figure 2 provides the result of our assessment, in which blue bars present the total number of errors, red bars present the total number of errors corrected by ChatGPT, X-axis represents error types, and Y-axis number of errors. It shows in the figure 2 that ChatGPT successfully identified and corrected all the errors, indicating it can be a valuable tool for assisting dental students in writing.

### Accuracy of ChatGPT responses

The ability of LLMs such as ChatGPT to consistently provide accurate responses to questions is vital in dental education and practice, because this cannot only help identify dental issues in an effective manner, but also enhance the applications and adoptions of LLMs in dentistry. On the other hand, inaccurate responses can create an image of untrustworthy or even be devastating, as precise and accurate results work as the key in clinical applications.

To measure the accuracy of ChatGPT’s responses, we formulated a rubric, which is described in table 2, to associate each criterion for evaluating ChatGPT responses with a grade. For simplicity, the scale of the rubric ranges from 0 to 3, with 0 indicating that a statement is not reflected in a ChatGPT output at all, grade 1 indicating a statement-related information is present but with discrepancies, 2 indicating slightly changed or altered wording in a ChatGPT response, and scale 3 indicating a precise statement is found in a ChatGPT response (Table 2).

To make our work reproducible by peers, we used a selected set of published articles instead of confidential clinical notes as test data for this assessment. Table 3 shows the main topics of four recently published papers (1^st^ column) [10,11,18,19]. Take the article 1 in the table as an example [10], which provides an overview of the implications of ChatGPT for dental medicine. For each key point in this paper (1^st^ column), we asked ChatGPT to summarize authors’ opinion. An abbreviated version of ChatGPT’s responses was provided in the 2^nd^ column of the table, so readers can easily compare with the key points in the 1^st^ column.

The rubric defined in table 2 was applied to grade ChatGPT’s responses. Our evaluation of ChatGPT’s responses was then provided in the last column of table 3, which shows that ChatGPT received a grade 2 (or 2/3=66.67%) for most of the questions about this article. It indicates that its ability to comprehend, accurately extract, and synthesize information from this document still needs to be improved.

The four articles in table 3 cover a variety of topics about ChatGPT: benefits, concerns, scientific discovery and innovation, etc. For ChatGPT’s summary of the key points of these four articles, the highest rubric score is 2 (or 2/3=66.67%), the lowest one is 0, and the median score is 2 (or 66.67%). The overall accuracy of 66.67% and its lack of consistency across these articles indicate that ChatGPT is better used with caution when applied to appraise literature. This result is consistent with a recent study by Ali et al. [13], who observed that ChatGPT provided accurate responses to majority of knowledge-based assessments, while consistently underperformed for critical appraisal of literature [13].

### Assistance in test preparation

Testing is a critical part of teaching and learning in dental education. We found ChatGPT can conveniently generate test questions for students (and instructors alike) on a variety of given topics. For example, for a request, “Can you generate 12 multi-choice questions about metagenomics & metatranscriptomics in dentistry”, it took ChatGPT only seconds to construct 12 quiz questions that include various question types, e.g., definition, application, and comparison, that can be adjusted based on difficulty levels: beginner, intermediate, or advanced. This is valuable and timesaving as it provides a good starting point for instructors to further refine based on context.

In dental education, the INBDE Dental Board Exam is one of the most important steps on a student’s journey to becoming a dentist. Here, INBDE, which stands for National Board Examinations, was designed to assist dental boards in determining the qualifications of those seeking licensure to practice dentistry or dental hygiene. To evaluate if ChatGPT can assist dental students in testing preparation, we asked ChatGPT to construct a test question for each INBDE code from a peer review resource. Its output was then evaluated by domain experts to confirm its relevance. Table 4 shows some example INBDE Test Objectives for dental examination and ChatGPT generated questions for them. As shown in table 4, the subject areas in this assessment are broad and compressive. It covers various aspects of the dental education in MMC’s SOD, including manual instructions for class 1 amalgam cavity preparation in an operative dentistry class, analysis of objectives for a nutrition class, study questions from a periodontics class, interpretation of dental radiographs for a dental radiography class, and analysis of chapter summary topics from a pathology class textbook, etc.

The analysis of ChatGPT generated questions showed that it is capable of bringing diverse information together on specific subjects in dentistry. In addition, some constructed questions were inappropriate to be used directly for testing if without refinement, indicating that the version 3.5 of ChatGPT still lacks higher-order intelligence that is required to correctly synthesize information. Despite its limitation, the questions and answers created using ChatGPT still have some values, in particular when used under guidance of domain experts or combined with authoritative references. Some could potentially help students identify subject areas that require additional efforts in their preparation for the INBDE test.

### Other assessments

We also conducted other assessments of ChatGPT. For example, we asked ChatGPT about dental health benefits of dietary fibers. ChatGPT responded with some recommendations as key benefits of dietary fibers: they promote regularity, reduce risk of colon cancer, help manage blood sugar, and lower cholesterol level, etc. Additionally, for a question about a dentistry operation procedure about class 1 amalgam preparation, ChatGPT response is very close to a dental expert recommendation in the area. ChatGPT summarized that class 1 amalgam preparation is a routine restorative procedure in operative dentistry and the key to a successful restoration is careful cavity preparation and amalgam condensation, followed by careful carving and polishing of the restoration.

## Discussion

In this paper, we explored the efficacy and potential applications of large language model (LLM)-based AI tools, in particular ChatGPT, in dental education. Our results demonstrated that ChatGPT can effectively recognize and correct dental terminologies and hence is a valuable tool for aiding dental students in writing. A potential limitation in our approach is that our assessment relied on manual evaluations, introducing an element of subjectivity, as different assessors may tend to have varying interpretations of errors. This variability could affect the accuracy and consistency of the error analysis. Furthermore, this study focused specifically on five categories of errors. Other types of errors or linguistic aspects that could influence writing quality were not considered. Therefore, a comprehensive analysis is still needed for future scale-up studies.

Furthermore, similar as other language models, ChatGPT may inherit biases in its training data. These biases can influence its comprehension and correction of errors, potentially resulting in inconsistencies or inaccuracies in the suggestions it offers. It may also have inherent limitations in its grasp of complex grammar rules, contextual nuances, or domain-specific language. These limitations could impede its ability to accurately identify and rectify errors, particularly in complex cases or instances involving uncommon error types.

The scope of this study needs to be expanded to encompass a longitudinal assessment of ChatGPT’s sustained impact on student performance and learning outcomes. It is also imperative to delve into the ethical considerations entailed in the adoption of AI tools, including ChatGPT, within educational contexts. Additionally, the exploration of the potential integration of ChatGPT into established dental education frameworks and platforms holds promise for enhancing practicality and accessibility for students. Another intriguing avenue for further investigation lies in comparing ChatGPT with conventional methods commonly employed in dental education, such as grammar and spelling correction tools.

Collecting feedback from a large cohort of dental students who have actively employed ChatGPT as an educational tool can yield invaluable insights into their individual experiences and perceptions. Additionally, gaining an understanding of the various ways students utilize ChatGPT, as well as their attitudes and overall satisfaction with the tool’s assistance, could inform meaningful enhancements for the future. By embarking on these areas of investigation, we can gain deeper insights into the potential benefits, limitations, and implications associated with the integration of ChatGPT in dental education.

## Conclusion

The recent rise of LLM-based AI chatbots, exemplified by ChatGPT and Bard, presents both opportunities and challenges in contemporary dental education. The pedagogical aspect of dentistry in the School of Dentistry (SOD) at Meharry Medical College predominantly revolves around lecture-based courses, where instructors disseminate knowledge through presentations and discussions. To address the challenges posed by the AI chatbots, this paper explored the potential utility and effectiveness of integrating ChatGPT into the pedagogical aspect of the dental education in the SOD.

In our evaluation of ChatGPT’s suitability for aiding dental students in writing, we observed its capability to effectively recognize and accurately rectify special dental terminologies, which require dental background for comprehension. This observation indicates ChatGPT can be a valuable writing assistant to help dental students with essay writing. Furthermore, we measured the aptitude of ChatGPT in responding to questions that we posed for a selection of published articles. Our assessment, based on a grading rubric, revealed a median accuracy of 66.7%. This suggests that while ChatGPT can extract and synthesize information from documents, there is room for improvement in its precision. We also investigated ChatGPT generated test questions to gauge their utility in assisting dental students in test preparation. We found some ChatGPT generated questions held potential value for students.

It is important to acknowledge that the manual assessments employed in our study have imposed a limit on the scale of this pilot study. Notwithstanding this limitation, we believe that this work provides important information that would be vital for future scaling-up studies.

## Figures and Tables

**Table 1:** Rubrics for evaluating ChatGPT responses to questions.

Error type	Examples incorrect terminologies provided to ChatGPT	Corresponding correct terminologies
Incorrect order	MDO, BO, DMO, BDO	MOD, OB, MOD, DOB
Capitalization & abbreviation	bw-xray	BWX
Capitalization	mod, fmx, Unc-15 probing instrument, Pa xray, pan xray	MOD, FMX, UNC, PA, PAN
Numerals instead of Roman numerals	Class 5 amalgam prep, Class 2 composite, Periodontics Stage 3, Mobility: Grade 1, Class 2 mobility	Class V, Class II, Stage III, Grade I, Class II

**Table 2:** Rubrics for evaluating ChatGPT responses to questions.

Grade	Scale Criteria	Description
3	Precise Statement	Verbatim statement without alteration or deviation.
2	Altered Wording, Same Meaning	Different wording but retains the original intent and meaning.
1	Related but with Discrepancies	Vague, adds extra info, partially relevant, or omits some original information.
0	Not Presented in the ChatGPT	Statement did not exist at all.

**Table 3:** Assessing the accuracy of ChatGPT’s summary of a published article [10,11,18,19].

Main topics in the article	Summary of ChatGPT’s response	Rubric value / accuracy (%)
**Article 1** ChatGPT’s implications for dental medicine		
LLMs for patient info & diagnosis	Helps describe symptoms & history	2 / 66.67%
LLMs for scalable telemedicine	Convenient & cost-effective access	2 / 66.67%
LLMs for real-time translation	Information not found	0 / 0.00%
ChatGPT not for medical guidance	Not the most suitable tool	2 / 66.67%
ChatGPT for insurance preauthorization	Verifies insurance info	2 / 66.67%
LLMs in dental schools	Personalizes learning 24/7	1 / 33.33%
**Article 2** Benefits of ChatGPT		
Personalized learning for individual student needs	Personalized experiences for students	2 / 66.67%
Freeing up educators to focus on important issues	Improved efficiency and productivity for educators	1 / 33.33%
**Article 3** Accelerated Scientific Discovery and Innovation		
Access to wide range of educational resources	Enhanced accessibility to education	2 / 66.67%
**Article 4** Concerns about ChatGPT		
AI biases and discrimination	Bias potential in AI algorithms	2 / 66.67%
Decrease in human interaction and critical thinking	Overreliance on AI hurts critical thinking	2 / 66.67%
Plagiarism	Data privacy and ownership concerns	1 / 33.33%
AI’s effectiveness in generating research papers	-	0 / 0.00%
Ethical concerns: transparency, accountability	Lack of transparency and accountability	1 / 33.33%
**Median**		**2 / 66.67%**

LLM: Large Language Model

**Table 4:** INBDE test questions generated by ChatGPT.

INBDE Test Objective	Peer-Review Resource	Example Question
CC#04 - Use clinical and epidemiological data to diagnose and establish a prognosis for dental abnormalities and pathology	Journal of Oral Pathology and Medicine	How can oral pathologists use molecular methods to improve the diagnosis and prognosis of oral cancer?
CC#05 - Recognize the normal range of clinical findings and distinguish significant deviations that require monitoring, treatment, or management	Journal of Dental Research	How do gingival crevicular fluid biomarkers differ in healthy patients compared to those with periodontal disease?
CC#06 - Predict the most likely diagnostic result given available patient information	Journal of Endon tonics	Can radiographic findings predict the success of non-surgical endodontic treatment?
CC#07 - Interpret diagnostic results to inform understanding of the patient’s condition	Journal of Prosthetics Dentistry	How can cone bear computed tomography be used to interpret implant placement and its effects on adjacent teeth?
CC#10 - Select the diagnostic tools most likely to establish or confirm the diagnosis	Journal of Periodontology	What is the most effective diagnostic tool for identifying periodontitis in its early stages?
CC#12 - Formulate a comprehensive diagnosis and treatment plan for patient management	Journal of Oral and Maxillofacial Surgery	What is the recommended treatment plan for a patient with a mandibular fracture?
CC#13 - Discuss etiologies, treatment alternatives, and prognoses with patients so they are educated and can make informed decisions concerning the management of their care	Journal of American Dental Association	What are the potential complications of a root canal procedure, and how can they be addressed?
CC#22 - Prevent, diagnose, and manage periodontal diseases	Journal of Clinical Periodontology	What are the most effective non-surgical periodontal treatments for managing periodontitis?
CC#41 - Evaluate scientific literature and integrate new knowledge and best research outcomes with patient values and other sources of information to make decisions about treatment	Journal of Evidence-Based Dental Practice	How can clinicians use evidence-based dentistry to make treatment decisions for patients with temporomandibular joint disorders?
